# Quantification of overall tumor burden using longitudinal magnetic resonance imaging improves response assessment in orthotopic murine hepatocellular carcinoma models

**DOI:** 10.1038/s41598-026-38125-2

**Published:** 2026-02-05

**Authors:** Isabella Lurje, Wiebke Werner, Nicole Hilbert, Ajay-Mohan Mohan, Kirsten Reers, Anne Schlutt, Maria Kuzminskaya, Yaroslava Shevchenko, Leke Wiering, Ines Eichhorn, Winfried Brenner, Nicola Beindorff, Frank Tacke, Linda Hammerich

**Affiliations:** 1 Department of Hepatology and Gastroenterology, Campus Charité Mitte and Campus Virchow-Klinikum, Charité - Universitätsmedizin Berlin, Berlin, Germany; 2 Department of General, Visceral and Transplantation Surgery, Heidelberg University Hospital, Heidelberg, Germany; 3 Department of Nuclear Medicine, Charité-Universitätsmedizin Berlin, Berlin, Germany; 4 Berlin Experimental Radionuclide Imaging Center (BERIC), Charité-Universitätsmedizin Berlin, Berlin, Germany; 5 German Consortium for Translational Cancer Research (DKTK), Partner Site Berlin, Berlin, Germany; 6 National Center for Tumor Diseases (NCT), Partner Site Berlin, Berlin, Germany

**Keywords:** Cancer, Oncology

## Abstract

Hepatocellular carcinoma (HCC) is one of the most frequent causes of cancer-related death worldwide. Mirroring the complexity of human HCC with its underlying liver disease requires multilocular orthotopic tumor models in mice, where objective tumor quantification in vivo is challenging, especially longitudinally. We investigated magnetic resonance imaging (MRI) to noninvasively quantify orthotopic HCC in an immunotherapy setting. Orthotopic HCCs were induced in diethylnitrosamine (DEN)-injected mice with either CCl_4_-induced hepatic fibrosis or dietary metabolic dysfunction-associated steatotic liver disease (MASLD) to mirror the most frequent etiologies. Growth kinetics over the course of immune checkpoint inhibitor treatment with anti-Programmed Death Ligand 1 (αPD-L1) were modeled by measuring the overall tumor burden (OTB) using MRI and compared to untreated animals. Tumor parameters, such as tumor volume, liver weights at sacrifice and largest tumor diameter were analyzed. We demonstrate that MRI is a reliable imaging tool for both pretreatment tumor confirmation as well as the longitudinal quantification of overall tumor burden under investigational treatment. While measuring only the largest tumor diameter yielded significant differences between αPD-L1-treated animals and untreated controls in the long-term setting, these trends in tumor response were not confirmed by MRI-based OTB measurement. In MASLD-HCC, tumors did not respond to PD-L1 blockade, thus confirming the OTB data and reflecting the immunotherapy resistance observed in the human setting and highlighting the translational relevance of the model. Orthotopic fibrosis-HCC and MASLD-HCC mouse models can be enhanced through the longitudinal use of MRI while reducing endpoints and animal numbers. The DEN-CCl_4_ and DEN-Western Diet models mirror the αPD-L1 response from the human setting and require OTB measurement, because largest diameters may underestimate the total tumor mass in orthotopic HCC.

**Supplementary Information:**

The online version contains supplementary material available at 10.1038/s41598-026-38125-2.

## Introduction

Hepatocellular carcinoma (HCC) is a highly aggressive malignancy that usually arises against the background of chronic liver disease^1^. The most common etiologies worldwide include viral hepatitis, chronic excessive alcohol consumption, and obesity/metabolic syndrome^2^. While viral or alcohol-induced hepatitis contributes to the classical sequence of fibrosis to cirrhosis^3^, metabolic dysfunction-associated steatotic liver disease (MASLD) is characterized by steatosis and, later, inflammation when progressing to metabolic dysfunction-associated steatohepatitis (MASH). The latter HCC etiology has increased dramatically due to the worldwide obesity epidemic – currently, half of the deaths caused by liver cirrhosis and over a third of deaths caused by HCC in the USA are attributed to MASLD^4^. While clinical treatment decisions are primarily influenced by tumor extent and liver function^5^, recent evidence points towards significant prognostic, immunological and molecular differences between different tumor etiologies, such as MASLD-HCC and fibrosis/cirrhosis-HCC^6,7^. Since 2020, therapeutic first-line options for unresectable HCC include an anti-programmed death ligand 1 (αPD-L1) antibody combination treatment^8^.

The HCC tumor microenvironment (TME) is central to immune evasion under checkpoint immunotherapy^9^. Particularly MASLD-HCC may respond less well to checkpoint inhibitor therapy, which may result in less pronounced survival benefits in patients with MASLD-HCC undergoing checkpoint inhibitor treatment^10^. Thus, sophisticated mouse models that mirror key aspects of the underlying chronic liver disease with its tumor etiology and TME^11^ are essential to the investigation of hepatocarcinogenesis, cell-cell interactions, and to the preclinical testing of potential therapies for HCC.

Tumor models closely recapitulating human disease, and thus bearing high translational value, include transgenic mice and environmentally induced models in wild-type mice^12^. The latter includes diethylnitrosamine (DEN)-injection-based HCC, which results in reproducible, spontaneous, multilocular liver tumors. The injection of the highly mutagenic agent in 2-week old mice can furthermore be combined with dietary interventions or hepatotoxins to model MASH-HCC or a fibrosis-HCC phenotype, resulting in a closer similarity to human etiology-dependent pathogenesis^12,13^. Due to the multilocularity of disease, analyzing the extent of tumor formation can present a challenge, especially when quantifying tumor load to compare the extent of disease between treatment groups. Unlike in models with tumor cell inoculation, the multilocular mutational effects of DEN result in mice bearing 20–40 hepatic tumors. Reported methods of tumor quantification include diameter and count of tumors on the liver surface^14^, measuring the largest tumor diameter, or employing surrogate parameters of tumor growth, like liver weight or liver-to-bodyweight ratio^15^. These parameters represent single endpoint parameters acquired after euthanasia and do not allow determination of growth kinetics of individual nodules. Potential dilemmas in non-standardized reported endpoints in preclinical research include the impaired comparability between studies and the selective reporting of findings consistent with the researchers’ hypothesis, for example only of selected tumor quantification parameters. Furthermore, dietary models severely alter liver and overall body weight, impairing the comparability of both liver weight and liver-to-bodyweight ratio between etiological HCC models^16,17^.

Here, we demonstrate that imaging murine fibrosis-HCC and MASH-HCC with magnetic resonance imaging (MRI) and calculating the lesion volumes by a volume of interest (VOI)-based assessment method, objectively quantifies the multilocular overall tumor load. We validate this method in immune checkpoint-treated animals with fibrosis-HCC and MASLD-HCC etiologies in a longitudinal setting, circumventing the need to euthanize animals at multiple timepoints before humane endpoints are reached. Importantly, we demonstrate that conventional approaches may overestimate longitudinal changes in tumor load that are reproducibly induced by immune checkpoint inhibitor therapy, while MRI-based volume assessment allows calculation of the hepatic tumor load, without the need of additional endpoints.

## Methods

### HCC animal models

**Fig. 1:**
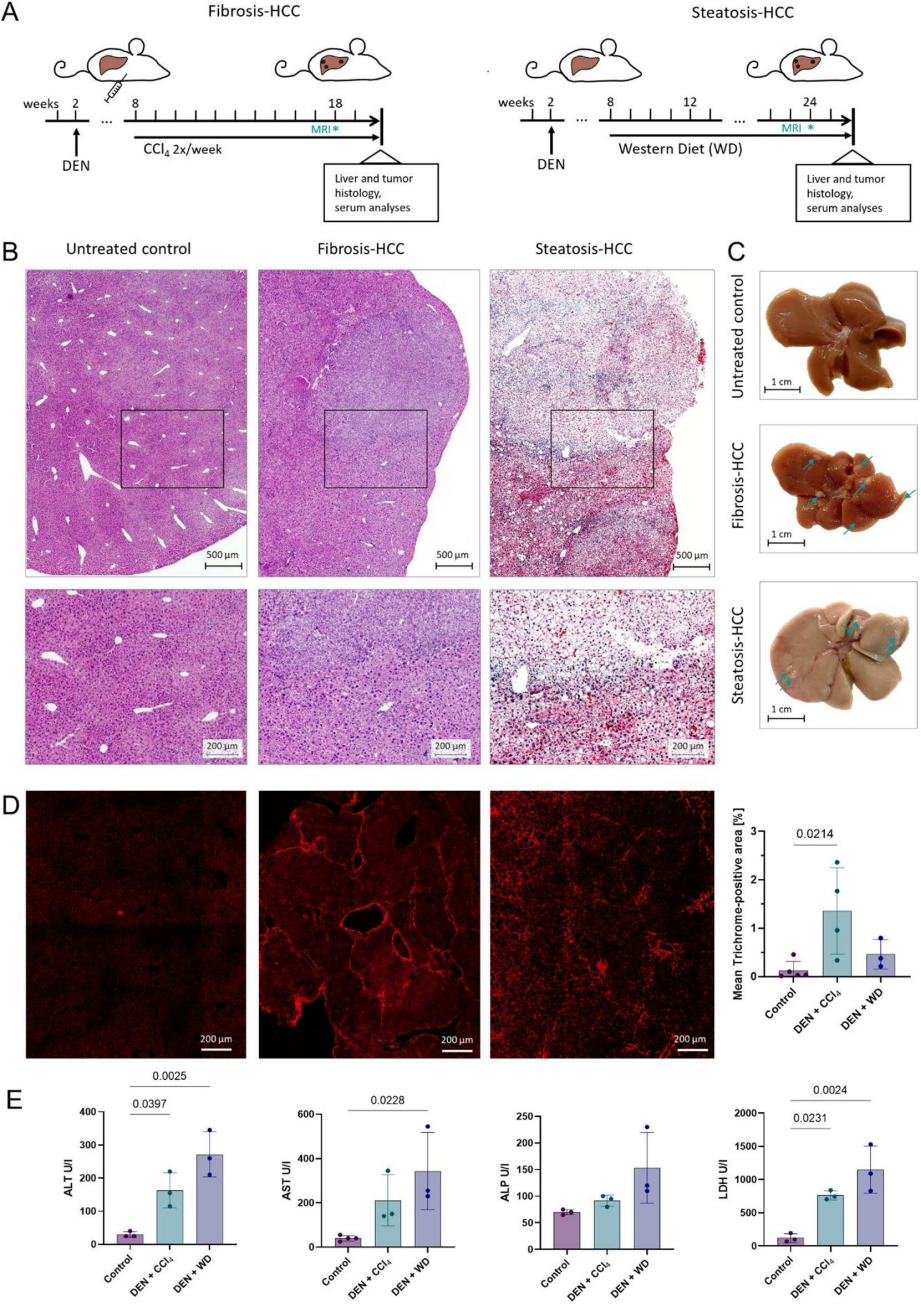
Experimental models of fibrosis- and MASLD-HCC show distinct histopathological and biochemical characteristics. (**A**) Experimental design of the fibrosis-HCC model (DEN + CCl_4_) and the MASLD-HCC model (DEN + WD). Mice were treated with DEN at 2 weeks of age and subjected to chronic liver injury via weekly CCl_4_ injections or Western Diet (WD) feeding starting at 8 weeks of age. Presence of HCC was confirmed with MRI scans at 18 and 24 weeks, respectively, and mice were euthanized at 20 and 26 weeks, respectively, for histological and serum analyses (**B**) Representative H&E-stained liver sections from untreated control, fibrosis-HCC and steatosis-HCC. Scale bars: top row = 500 μm, bottom row = 200 μm. (**C**) Macroscopic appearance of livers, scale bar: 1 cm. Arrows indicate tumor nodules. (**D**) Representative images of liver sections stained with Trichrome and visualized under polarized light for collagen deposition, scale bar = 200 μm. (**E**) Serum levels of liver injury markers (ALT, AST, ALP, LDH) at endpoint. Data are presented as mean ± SD; *n* = 3–5 per group. One-way ANOVA with post hoc testing. Abbreviations: ALP, alkaline phosphatase, ALT, alanine aminotransferase, AST, aspartate aminotransferase, CCl_4,_ carbon tetrachloride, DEN, diethylnitrosamine, HCC, hepatocellular carcinoma, H&E, Hematoxylin and Eosin, LDH, lactate dehydrogenase, MASLD, metabolic dysfunction-associated steatotic liver disease.

All experiments were approved by the Berlin State Office for Health and Social Affairs (LAGeSo, approval no. G0310/19) and study was performed in accordance with German animal protection law. Study planning followed the PREPARE guidelines (https://norecopa.no/PREPARE) and study results are reported in accordance with the ARRIVE guidelines (https://arriveguidelines.org/). Male wildtype C57BL/6 mice were obtained from the internal breeding colony at Charité – Universitätsmedizin Berlin, bred in-house and housed in filter top cages under a 12-hour light/dark cycle and ad libitum access to food and water. Cages were equipped with wood shaving bedding and standardized enrichment (igloos, lofts, handling tunnels, nesting material, gnawing wood). Animals were monitored daily using scoring sheets in accordance with the OBSERVE guidelines (https://www.nature.com/articles/s41596-024-00998-w) and weighed weekly. Pups were injected with DEN (25 mg/kg bodyweight (BW) in 0.9% NaCl) intraperitoneally at 2 weeks of age and randomly assigned to treatment groups. From 8 weeks of age, the animals either underwent carbon tetrachloride (CCl_4_) injections (intraperitoneally twice per week, 0.5 ml/kg BW in corn oil) and remained on chow diet or were fed exclusively with Western-type diet (WD), with 21% butter fat and 1.25% of cholesterol (#E15723-34 Ssniff Spezialdiäten GmbH, Soest, Germany) to model liver fibrosis or MASLD, respectively. At 18 weeks in the DEN+CCl_4_ model, and 24 weeks in the DEN + WD model, very early tumors were detected with MRI. Two weeks after tumor confirmation with MRI, mice in the checkpoint group began receiving αPD-L1 (Clone 10 F.9G2, BioXcell, Lebanon, New Hampshire, USA) for their established tumors intraperitoneally every 3 days (100 ug at 2 mg/ml in 0.9% NaCl) (Fig. 1A). A subgroup of control mice without any liver disease (*n* = 5), and with DEN+CCl_4_ (*n* = 4)/ DEN + WD (*n* = 3) (at 20, 20 and 26 weeks of age, respectively) were euthanized for histologic characterization of the models. CCl_4_-treated mice were euthanized 3 days after the last CCl_4_ injection.

### Imaging and humane endpoints

At week 18 in the DEN+CCl_4_ HCC-fibrosis model and at week 24 in the DEN + WD HCC-MASLD model (which represent the time points that tumors first become consistently detectable in each model), mice underwent first imaging with MRI. Scans were acquired on a 3 Tesla MRI (MRS 3047, MR Solutions, Guildford, UK) with a vinyl methyl silicone rubber mouse body coil (MVQ, S/N: MVQ30074, MR Solutions) under isoflurane anesthesia (2.5-3.0%) with 0.7% oxygen at a flow rate of 0.7 l/min. Mice were placed in prone position on a heated (32 °C) single mouse bed (Equipement Vétérinaire Minerve, France) accounting for the additional heating generated in the coil. A respiratory pillow was used to monitor respiratory rate for anesthesia adjustment and for respiratory gating throughout image acquisition to minimize motion artefacts.

All MRI scans for tumor size and volume assessment were performed in axial orientation. The sequences for T1-weighted images had the following parameters: 256 × 256 × 42 matrix with dimensions 0.2 × 0.2 × 0.6 mm, repetition time (TR) 1134–1587 ms, 40–60 slices with 4 averages, and the fast T2-weighted spin-echo sequences had the following: 256 × 256 × 42 matrix with dimensions 0.2 × 0.2 × 0.6 mm, TR 3000–5000 ms, 36–50 slices with 4 averages.

MR imaging was performed every other week until animals reached humane endpoints and were euthanized with isoflurane overdose (Fig. 1A). Humane endpoints included: indicators of lasting pain (more than 24 h after analgesia), severely impaired overall condition (weight loss > 20%, palpable tumors > 10 mm, centralized circulation, lack of reaction to stimuli), or largest tumor diameter (of a single nodule) of 15 mm on MRI imaging.

### Image analysis

MRI images were analyzed with the Preclinical Scan interface (MR Solutions) to determine the largest tumor diameter and the PMOD 3.5 software (Bruker preclinical imaging, Fällanden, Switzerland) for overall tumor burden (OTB). The volume of interest (VOI) of each tumor was determined by manually contouring a region of interest (ROI) in each slice, which are summed up across slices and multiplied by the slice thickness by PMOD. All ROIs were drawn by the same investigator to avoid bias through interobserver variability and spot checked for accuracy by a second investigator. OTB was defined as the cumulative VOI of all hepatic tumor nodules across all acquired slices.

### Sample processing

Mice were euthanized using isoflurane overdose. Blood was drawn from the left heart ventricle into serum gel tubes and centrifuged for 10 min at 13,000 g, the serum layer was transferred into cryotubes and snap-frozen. Measurement of serum parameters, including ALT, AST, ALP and LDH, was performed by the central laboratory of Charité—Universitätsmedizin Berlin, Labor Berlin—Charité Vivantes using standardized certified assays. After transection of the portal vein, the liver was perfused with 10 ml DPBS (1x, Thermo Fischer Scientific, Waltham, MA, USA) through the left heart ventricle. Liver samples were harvested, fixed in 10% formalin (Sigma-Aldrich, St. Louis, MO, USA) and embedded in paraffin. Blocks were sectioned at 2 μm, mounted on glass slides and processed for immunohistochemistry or immunofluorescence. Unfixed liver samples were also frozen in cryomolds in Tissue Tek (Sakura Finetek USA, Torrance, CA, USA) in the vapor phase of liquid nitrogen and sectioned at 10 μm for Oil red O staining and 7 μm for immunofluorescence.

### Immunohistochemistry

Hematoxylin/eosin (H&E) staining was performed as previously described^18^.

For Masson´s trichrome staining, formalin-fixed paraffin embedded (FFPE) sections were deparaffinized at room temperature for 10 min each in two changes of ROTI^®^Histol (Carl Roth GmbH, Karlsruhe, Germany), then rehydrated through descending ethanol concentrations (2 changes of 100% ethanol, 5 min each, followed by 5 min in 96% ethanol and 5 min in 70% ethanol) and washed twice with dH_2_O for 5 min. Sections were stained sequentially, according to manufacturer’s instructions, with the Masson’s trichrome staining kit (Morphisto GmbH, Frankfurt am Main, Germany). Slides were dehydrated using ascending ethanol concentrations (5 min at 60% ethanol, 3 min each in 75% ethanol, 96% ethanol and two changes of 100% ethanol). Next, slides were cleared in two changes of xylene for 5 min each, and coverslipped with mounting medium. Images were acquired using the red autofluorescent spectrum of the collagen staining (Cy5 channel).

For Oil red O staining, cryosections were washed briefly in distilled water, then fixed in 4% formaldehyde (SAV Liquid Production GmbH, Germany) for 10 min and rinsed twice with water. Oil Red O working solution was prepared by diluting 30 mL of 0.5% Oil Red O in isopropanol (Sigma-Aldrich, Germany) with 20 mL of distilled water and filtering. Sections were stained for 20 min at room temperature, followed by three rinses in distilled water. Nuclei were counterstained with hematoxylin for 10 min and blued under running tap water. After air-drying for 2 h, sections were mounted using warm (50 °C) glycerol gelatin (Carl Roth) and coverslipped.

Images were acquired on a Zeiss Axio Observer 7 microscope (Carl Zeiss AG, Oberkochen, Germany) at 10x magnification using the ZEISS ZEN Blue edition software v.3.2. Stitching and shading correction were performed after acquisition. Quantification was performed by selecting non-tumorous areas and defining the fraction of positive tissue using Fiji/ImageJ^19^.

### Immunofluorescence

For immunofluorescent staining of CD8 and PD-1, FFPE sections were deparaffinized as described above, followed by antigen retrieval with universal heat-induced epitope retrieval agent solution (Abcam, United Kingdom) at 94 °C for 20 min and blocking of non-specific antibody binding with 2.5% normal horse serum (Vector Laboratories, USA) for 1 h at room temperature. After overnight incubation at 4 °C with rat anti-mouse CD8a (5 µg/ml, clone 4SM15, Thermo Fisher, USA) and goat anti-mouse PD-1 (1 µg/ml, polyclonal, R&D Systems, USA), secondary anti-rat IgG2a Alexa Fluor 647 (4 µg/ml, ab172333, Abcam, USA) and anti-goat IgG CF 750 (4 µg/ml, SAB4600444, Sigma-Aldrich, USA) antibodies were applied for 1 h at room temperature. Cell nuclei were stained with DAPI (20 µg/ml, Sigma-Aldrich, USA).

For immunofluorescent staining of PD-L1, cryosections were fixed in ice cold acetone, followed by blocking of non-specific antibody binding with 0.5% normal goat serum for 20 min. Sections were incubated at 4 °C overnight with rabbit anti-mouse PD-L1 (clone ARC2478, ABclonal, USA), followed by goat anti-rabbit IgG Alexa Fluor 647 (4414 S, Cell Signaling, USA) for 1 h at room temperature. Cell nuclei were stained with DAPI.

Image acquisition and post-processing was performed as described for histological staining at 20x magnification. Quantification was performed by selecting tumor regions, manually counting CD8 + PD-1 + cells and calculating the percentage of PD-L1 positive area with Fiji/Image J (version 2.15.0).

### Statistical analysis

Statistical analyses were performed using GraphPad Prism (v. 10.4.2, GraphPad Software, USA). Data are presented as mean ± standard deviation (SD). For comparison of 2 groups unpaired t-test with Welch´s correction was used. For comparison between multiple groups, one-way ANOVA was used. For comparison of longitudinal tumor growth data, a mixed-effects analysis with Geisser-Greenhouse correction and Šídák’s multiple comparisons test was used. p-values < 0.05 were considered statistically significant. For OTB and tumor diameter assessments over time, individual timepoints were compared between groups. All image-derived measurements were conducted in a blinded fashion to prevent bias. Sample sizes per group are indicated in figure legends or tables.

## Results

### The DEN-CCl_4_ and DEN-WD models replicate hallmarks of human disease

Hallmarks of human HCC include the occurrence of multiple, genetically diverse tumor nodules in either chronically inflamed fibrotic/cirrhotic (e.g. in alcohol-associated liver disease or viral hepatitis) or in steatotic (often non-cirrhotic) livers (i.e. MASLD-HCC)^1^. DEN-CCl_4_ treatment and DEN-WD feeding in mice resulted in the establishment of multilocular hepatic HCC at 18 and 24 weeks, respectively (Fig. 1A-C). Animals with DEN-CCl_4_ treatment had significantly higher levels of fibrosis, as evidenced by trichrome quantification (mean positive area [% of tissue] 0.1276 ± 0.1865 in controls versus 1.355 ± 0.8908, *p* = 0.0214), while the DEN-WD treatment resulted in a trend towards higher liver fibrosis in the non-tumorous area in comparison to controls (0.4608 ± 0.3019 *p* = 0.6933, Fig. 1D). Animals with MASLD-HCC showed a significantly higher accumulation of hepatic fat on Oil Red O staining than control animals (mean positive area [% of tissue] 1.068 ± 1.605 versus 30.17 ± 7.519, *p* = 0.0192, Supplementary Fig. 1). None of the animals had developed visible distant/extrahepatic metastases at the time of euthanasia.

Liver enzymes and injury readouts were measured in serum at the time of humane euthanasia. Both DEN-CCl_4_ and DEN-WD mice with early tumors had significantly higher alanine aminotransferase (ALT) levels than untreated age-matched controls (mean ALT 163.3 U/l, adjusted *p* = 0.0397 and 271.7 U/l, adjusted *p* = 0.0025 vs. 30.0 U/l, respectively) and significantly higher lactate dehydrogenase (LDH) levels (mean LDH 763.3 U/l, adjusted *p* = 0.0231, and 1150 U/l, adjusted *p* = 0.0024 vs. 121.7 U/l). Mean aspartate aminotransferase (AST) levels were higher in DEN-CCl_4_ (211.7 U/l) and DEN-WD (343.3 U/l) mice than in controls (40 U/l, *p* = 0.1826 and *p* = 0.0228, respectively), while only trends in alkaline phosphatase (ALP) levels were noted between groups (mean DEN-CCl_4_ 91.67 U/l, DEN-WD 153.3 U/l, controls 70 U/l, *p* = 0.7831 and *p* = 0.0881, respectively, Fig. 1E).

### Standardized sequences enable tumor visualization in fibrosis-HCC and MASLD-HCC

To follow up mice for tumor extent and growth pattern, we tested imaging conditions to fulfil the following prerequisites: (1) to detect small tumors in initial disease stages, (2) to consistently identify even heterogenous tumors, regardless of their morphological variability, (3) to account for the underlying liver disease and thus allow monitoring across progressing fibrosis and steatosis stages.

**Fig. 2:**
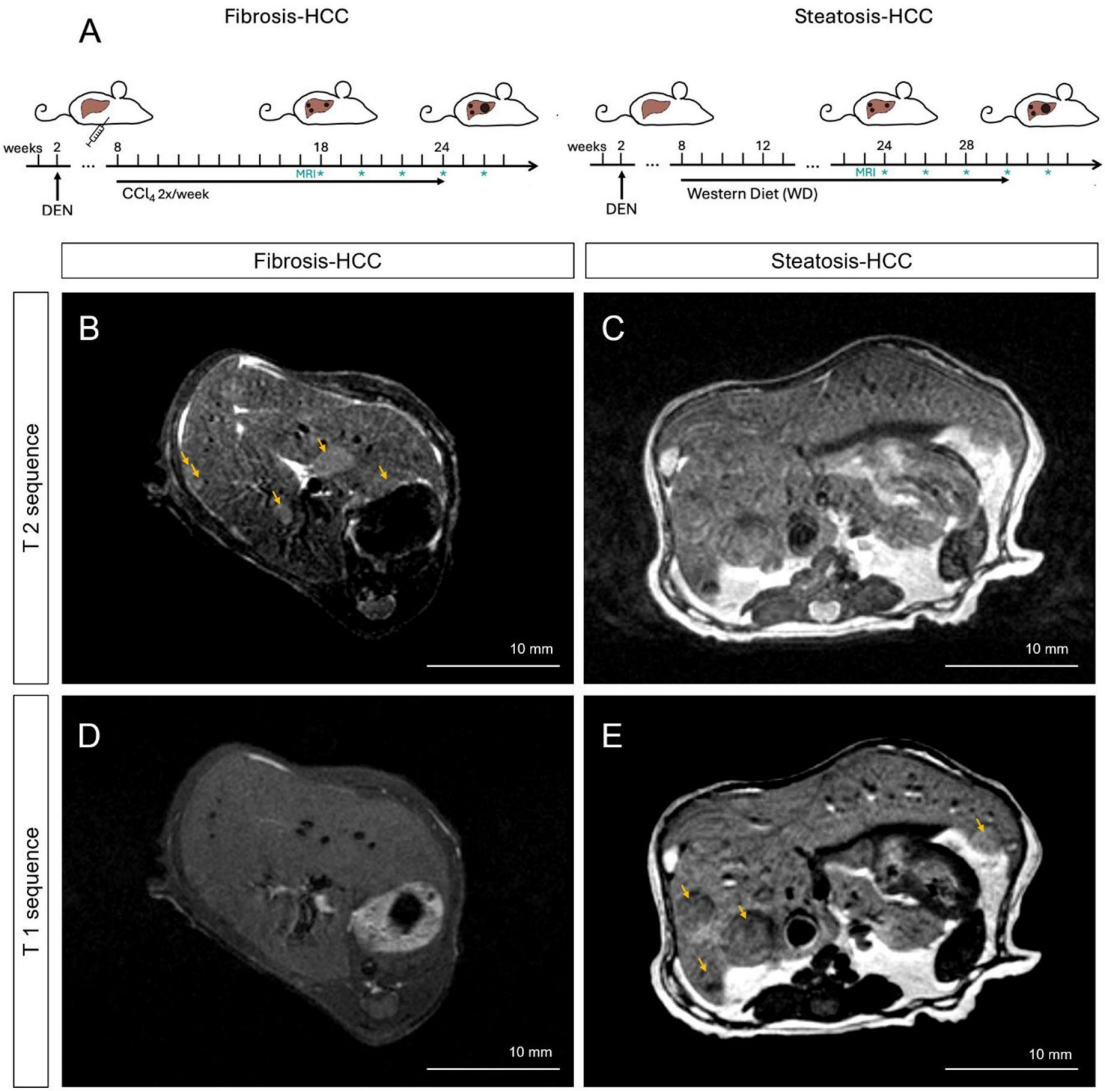
MRI enables non-invasive visualization of HCC in fibrosis- and steatosis-associated HCC mouse models. (**A**) Schematic overview of the experimental timelines for fibrosis-HCC (left) and steatosis-HCC (right) models. Mice were treated with DEN at 2 weeks of age and subjected to chronic liver injury via weekly CCl_4_ injections or WD feeding starting at 8 weeks of age. Tumor growth was monitored longitudinally with MRI. (**B**,**C**) Representative axial T2-weighted MRI images from mice in the fibrosis-HCC (**B**) and steatosis-HCC (**C**) groups. (**D**,**E**) T1-weighted MRI images of fibrosis-HCC (**D**) and steatosis-HCC (**E**) livers. Arrows indicate tumor nodules. Scale bar = 10 mm. Abbreviations: CCl4, carbon tetrachloride, DEN, diethylnitrosamine, HCC, hepatocellular carcinoma, MRI, magnetic resonance imaging, WD Western Diet.

In the fibrosis-HCC model, native T2-weighed (rendering water/edema hyperintense) sequences provided excellent soft tissue contrast, enabling reliable delineation of tumor nodules against the surrounding fibrotic parenchyma (Fig. 2B). Tumors typically appeared hyperintense relative to the liver, allowing for clear visualization of lesion borders and size assessment. In contrast, T1-weighted sequences (rendering fat hyperintense) yielded low contrast resolution, with poorly defined tumor boundaries (Fig. 2D). In MASLD-HCC, on T2-weighted images (Fig. 2C), tumors appeared isointense to only mildly hyperintense, limiting reliable identification due to the diffuse steatotic signal of the parenchyma. However, T1-weighted sequences (Fig. 2E) enabled effective lesion visualization and margin definition with hypointense tumor foci against the hyperintense steatotic parenchyma.

The DEN model is a genetically highly heterogenous model^12^. This was visibly reflected across longitudinal scans, where hypo- and hypervascular nodules were detected, but remained clearly demarcated in T2 sequences and were consistent in their visualization pattern (Supplementary Fig. 2).

### Response to checkpoint treatment in Fibrosis-HCC

**Fig. 3:**
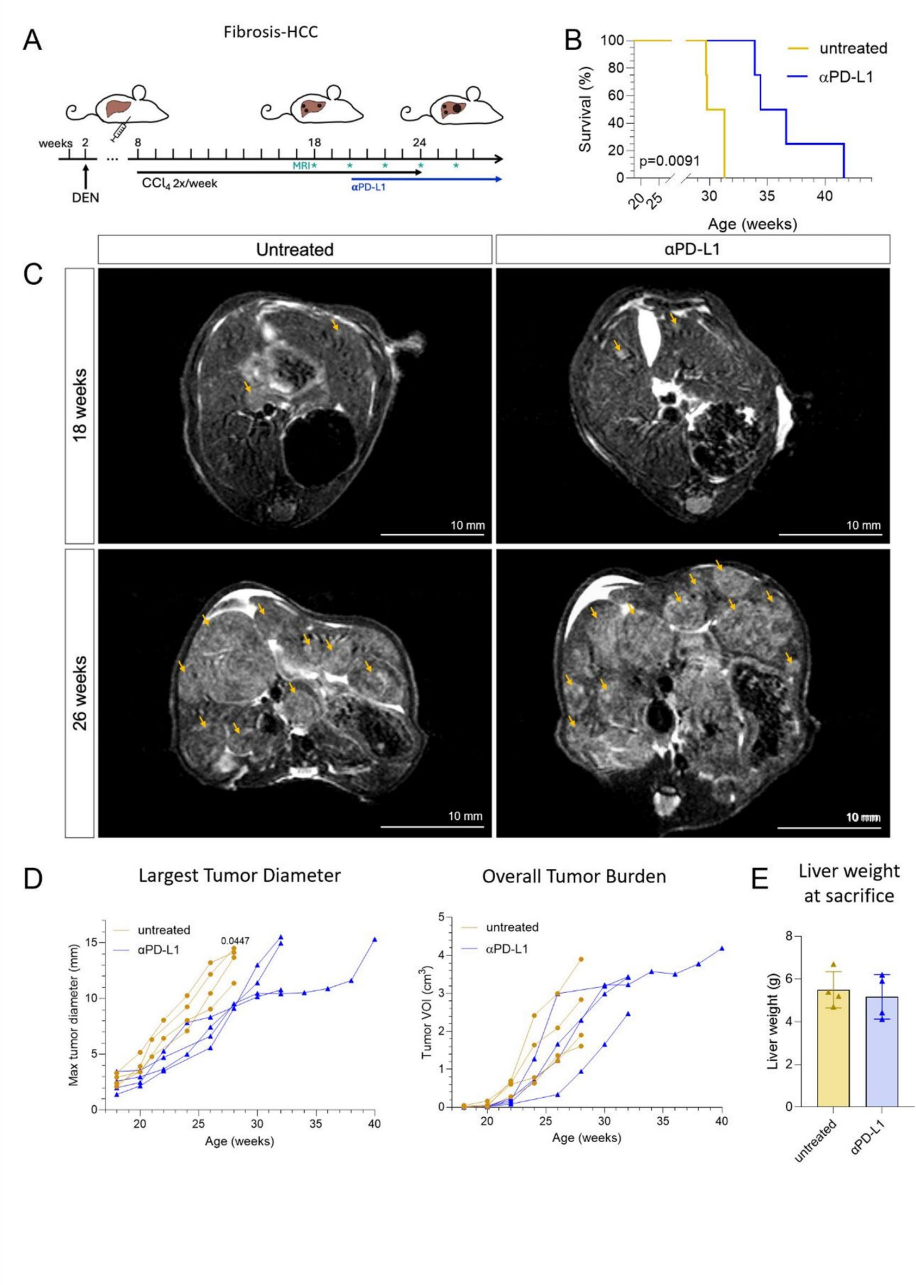
MRI-based monitoring of immunotherapy response in the fibrosis-HCC model. (**A**) Experimental design of the fibrosis-HCC model. αPD-L1 treatment was initiated 2 weeks after tumor confirmation at week 18, and MRI was performed bi-weekly to evaluate tumor burden. (**B**) Group-related survival. Log-rank (Mantel-Cox) test (**C**) Representative axial T2-weighted MRI images of untreated and αPD-L1–treated mice at 18 and 26 weeks of age. Arrows indicate tumor nodules. Scale bars = 10 mm. (**D**) Quantification of tumor progression using MRI, including the largest tumor diameter (left) and overall tumor burden (VOI, right) over time. Each line represents one mouse. Mixed-effects analysis with Geisser-Greenhouse correction and Šídák’s multiple comparisons test. (**E**) Liver weights at euthanasia. Data are presented as mean ± SD. Unpaired t-test with Welch´s correction. Only p values < 0.05 are annotated. Abbreviations: CCl_4_, carbon tetrachloride, DEN, diethylnitrosamine, HCC, hepatocellular carcinoma, MRI, magnetic resonance imaging, αPD-L1, anti-programmed death-ligand 1, VOI, volume of interest.

Since tumors in both models express PD-L1 and harbor PD-1 + CD8+ T cells (Supplementary Fig. 3), we tested the efficacy of therapeutic αPD-L1 application and followed tumor growth longitudinally. In the DEN-CCl_4_ model, mice were imaged every two weeks with T2 sequences (Fig. 3A). Mice in the αPD-L1 group survived significantly longer than untreated animals (30.6 weeks vs. 35.5 weeks of age, log-rank *p* = 0.0091, Fig. 3B), primarily because tumor size (largest tumor diameter) was the most common humane endpoint reached. Representative images show similar tumor sizes at baseline (18 weeks, mean 2.705± 0.5454 mm untreated versus 2.368 ± 0.8833 mm αPD-L1-treated, *p* = 0.9910), but a reduced maximal tumor size in αPD-L1-treated mice, compared to untreated controls in the fibrosis-HCC model at 26 weeks (Fig. 3C-D). Tumor diameters were reduced in the αPD-L1 group, (week 22: mean reduction ± SD:2.10 ± 0.79 mm, *p* = 0.1140; week 24: -2.238 ± 1.579 mm, *p* = 0.9084; week 26: -4.23 ± 1.09 mm, *p* = 0.0663; week 28: -4.10 ± 0.73 mm, *p* = 0.0447), with the 26 weeks timepoint reaching significance (Table 1). However, OTB, as determined by the tumor VOI, was not significantly affected across longitudinal timepoints (Fig. 3D; Table 2), indicating that measuring only 2D tumor diameters underestimates actual hepatic tumor mass. Relative changes from initial tumor size and volume did not show significant differences (Supplementary Fig. 3A). Liver weight at the humane endpoint was similar between untreated animals and the αPD-L1 group (*p* = 0.6371, Fig. 3E), further indicating that total tumor burden does not differ between groups. Accordingly, liver weight tended to associate more closely with MRI-derived tumor volume (VOI; *R* = 0.258, *p* = 0.537) than with tumor diameter (*R* = − 0.131, *p* = 0.717, Supplementary Fig. 3B).

**Table 1:** Largest tumor diameters on MRI.

Week	Untreated (mean ± SD)	*N*	α-PD-L1 (mean ± SD)	*N*	Difference (mean ± SD)	p*
Fibrosis-HCC						
18	2.705 ± 0.5454	4	2.368 ± 0.8833	4	− 0.3375 ± 0.5191	0.9910
20	3.985 ± 0.8328	4	2.798 ± 0.6081	4	− 1.188 ± 0.5156	0.3312
22	6.403 ± 1.340	4	4.300 ± 0.8495	4	− 2.103 ± 0.7934	0.1140
24	8.668 ± 1.381	4	6.430 ± 2.008	2	− 2.238 ± 1.579	0.9084
26	11.23 ± 1.843	4	6.998 ± 1.172	4	− 4.228 ± 1.092	0.0663
28	13.43 ± 1.406	4	9.325 ± 0.2899	2	− 4.103 ± 0.7321	**0.0447**
30		0	11.26 ± 1.283	4		
32		0	12.93 ± 2.693	4		
MASLD-HCC						
24	2.978 ± 0.7083	4	2.640 ± 0.8401	3	− 0.3375 ± 0.6006	0.9994
26	4.525 ± 1.751	4	3.967 ± 0.8084	3	− 0.5583 ± 0.9923	0.9994
28	6.205 ± 2.088	4	5.507 ± 0.7379	3	− 0.6983 ± 1.128	0.9988
30	7.520 ± 2.113	4	8.350 ± 0.6296	3	0.8300 ± 1.117	0.9962
32	9.395 ± 3.083	4	9.010 ± 0.0985	3	− 0.3850 ± 1.543	> 0.9999
34	11.06 ± 3.747	4	10.11 ± 1.177	3	− 0.9525 ± 1.993	0.9998
36	12.09 ± 3.780	3	11.90 ± 1.292	3	− 0.1900 ± 2.306	> 0.9999

*p given for mixed-effects analysis with Geisser-Greenhouse correction and Šídák’s multiple comparisons test. Significant p values are depicted in bold.

*HCC* hepatocellular carcinoma, *MASLD* metabolic dysfunction-associated steatotic liver disease, *MRI* magnetic resonance imaging, *PD-L1* programmed death-ligand 1, *SD* standard deviation.

**Table 2:** Overall tumor burden on MRI.

Week	Untreated (mean ± SD)	*n*	α-PD-L1 (mean ± SD)	*n*	Difference (mean ± SD)	p*
Fibrosis-HCC						
18	0.0212 ± 0.0218	4	0.0078 ± 0.00411	3	− 0.0134 ± 0.0112	0.8908
20	0.0673 ± 0.0680	4	0.0279 ± 0.0098	4	− 0.0394 ± 0.0344	0.9108
22	0.5546 ± 0.1867	4	0.1597 ± 0.06423	4	− 0.3950 ± 0.0987	0.1075
24	1.369 ± 0.8278	4	0.9908 ± 0.4073	2	− 0.3782 ± 0.5042	0.9836
26	1.921 ± 0.8146	4	1.562 ± 1.106	4	− 0.3589 ± 0.6870	0.9971
28	2.563 ± 1.033	4	1.621 ± 0.9526	2	− 0.9420 ± 0.8488	0.9381
30		0	2.766 ± 0.7448	4		
32		0	3.141 ± 0.4573	4		
MASLD-HCC						
24	0.1198 ± 0.1255	4	0.0281 ± 0.00996	3	− 0.0917 ± 0.0630	0.8539
26	0.2674 ± 0.1850	4	0.1963 ± 0.09988	3	− 0.0711 ± 0.1090	0.9959
28	0.6492 ± 0.4052	4	0.5919 ± 0.5312	3	− 0.0573 ± 0.3676	> 0.9999
30	1.004 ± 0.8710	4	0.8710	1	n.a.	
32	1.514 ± 1.031	4	1.031	1	n.a.	
34	2.626 ± 1.046	4	2.453 ± 0.7133	3	− 0.1729 ± 0.6657	> 0.9999
36	2.673 ± 0.4552	3	3.130 ± 0.1472	3	0.4574 ± 0.2762	0.8209

*p given for mixed-effects analysis with Geisser-Greenhouse correction and Šídák’s multiple comparisons test. Significant p values are depicted in bold.

*HCC* hepatocellular carcinoma, *MASLD* metabolic dysfunction-associated steatotic liver disease, *MRI* magnetic resonance imaging, *PD-L1* programmed death-ligand 1, *SD* standard deviation.

### Response to checkpoint treatment in MASLD-HCC

**Fig. 4:**
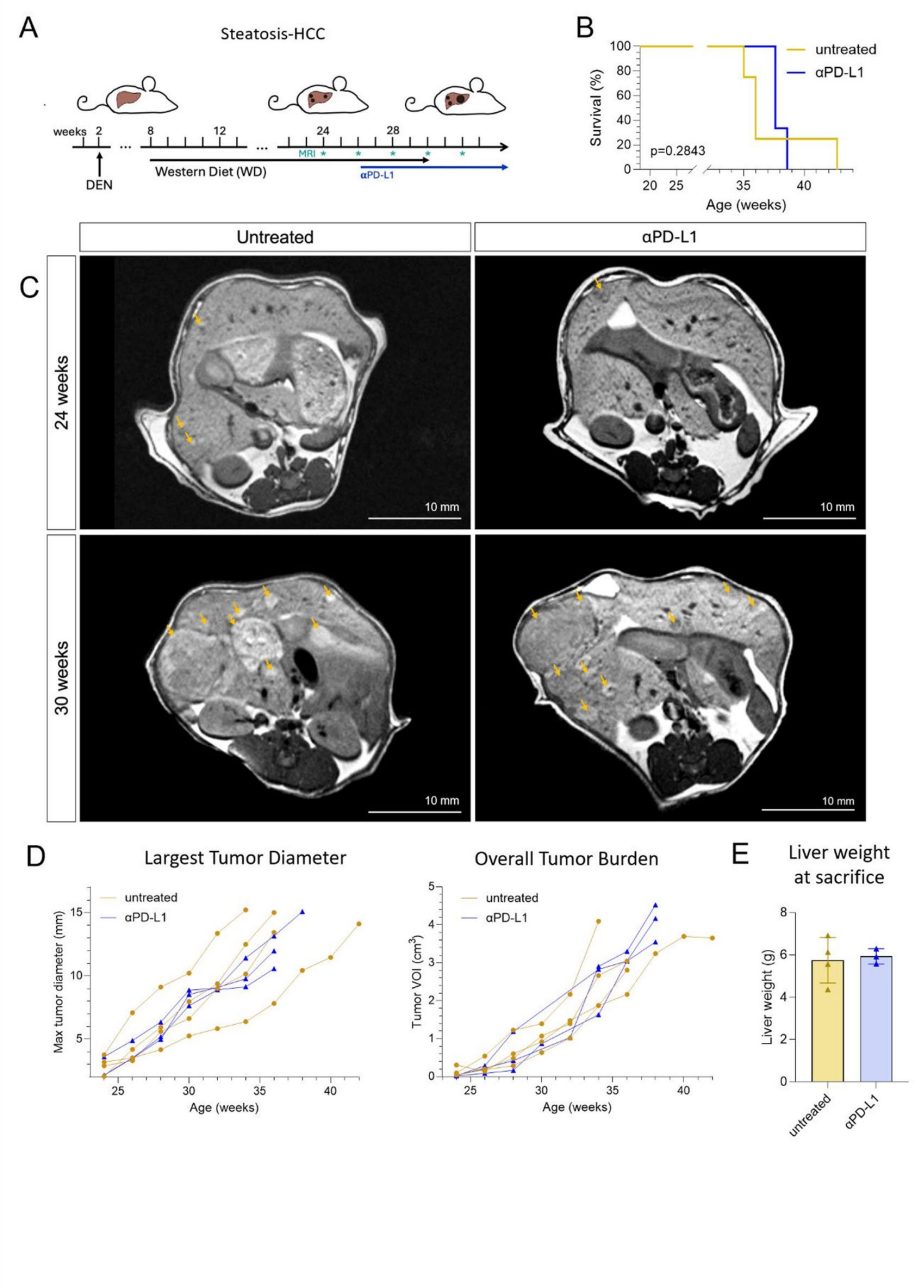
MRI-based monitoring of immunotherapy response in the MALSD-HCC model. (**A**) Experimental design of the steatosis-HCC model. αPD-L1 treatment was initiated 2 weeks after tumor confirmation at week 24, and MRI was performed bi-weekly to evaluate tumor burden. (**B**) Group-related survival. Log-rank (Mantel-Cox) test (**C**) Representative axial T1-weighted MRI images of untreated and αPD-L1–treated mice at 24 and 32 weeks of age. Arrows indicate tumor nodules. Scale bars = 10 mm. (**D**) Quantification of tumor progression using MRI, including the largest tumor diameter and the overall tumor burden (tumor volume of interest, VOI) over time. Each line represents one mouse. Mixed-effects analysis with Geisser-Greenhouse correction and Šídák’s multiple comparisons test. (**E**) Liver weights at euthanasia. Data are presented as mean ± SD. Unpaired t-test with Welch´s correction. Only p values < 0.05 are annotated. DEN, diethylnitrosamine, HCC, hepatocellular carcinoma, MRI, magnetic resonance imaging, αPD-L1, anti-programmed death-ligand 1, VOI, volume of interest, WD, Western diet.

To evaluate the therapeutic impact of PD-L1 blockade in the MASLD-HCC model, longitudinal T1-weighted MRI was performed every two weeks starting at 24 weeks (Fig. 4A). There was no significant difference in survival (36.0 weeks vs. 37.6 weeks of age, log-rank *p* = 0.6888, Fig. 4B). Representative axial scans illustrate comparable tumor presentation at baseline and during tumor progression (Fig. 4C). Overall, neither largest tumor diameters nor volumetric tumor burden (Fig. 4D), nor their relative change from baseline (Supplementary Fig. 3C) was significantly changed by αPD-L1 treatment in the MASLD-HCC model (Tables 1 and 2). Liver weight at humane endpoints was similar between groups (*p* = 0.7667, Fig. 4E) and did not significantly associate with MRI readouts (Supplementary Fig. 3D). Tumor growth kinetics between α-PD-L1 and untreated mice were largely overlapping, which is consistent with the known relative immunotherapy resistance in human MASLD-HCC^10^.

## Discussion

The majority of HCC arises against the background of chronic liver disease, predominantly fibrosis and steatosis. Tumor etiology confers distinct effects on the TME^20^, necessitating the use of immunocompetent orthotopic HCC models with concomitant chronic liver injury for the investigation of immunotherapy. For example, MASLD-HCC is driven and characterized by specific autoreactive mechanisms, including the dendritic cell-T cell axis, that are unique to this tumor etiology and inhibit the efficacy of immunotherapy^6,21^.

Compared to subcutaneous tumor models, the DEN-CCl_4_ and DEN-WD liver tumor reproduce the major etiological, histopathological, immune and transcriptomic hallmarks of human HCC. Unlike xenograft models, tumors arise *de novo* within the liver, and DEN-treated animals are fully immune-competent, allowing to study truly endogenous immune mechanisms in the TME. The combination of DEN with fibrosis or steatosis induction further shapes the liver environment towards the different etiologies of human chronic liver disease. Recently, DEN-CCl_4_ models were shown to exhibit proteomic and molecular features closely resembling human HCC, particularly the poor-prognosis subtype, thereby underscoring its suitability as a representative model for studying human HCC pathogenesis and progression^22^. Likewise, HCC models combining DEN injection with MASH inducing diets have been found to display similar expression patterns of both tumor-promoting and tumor-suppressing molecules as human steatosis-related HCC^23,24^. The DEN-WD model utilized here was recently identified to not only exhibit key histological features typical of MASH-HCC, but also to closely correspond to the transcriptomic phenotype of these tumors (Hoshida S1 subtype)^25^. These similarities underscore the translational relevance of both models, especially for immunotherapy studies. However, orthotopic models present challenges for tumor assessment due to their multifocal distribution, non-palpable growth, and localization within the body throughout the liver, which limits the feasibility of external measurement. The development of standardized longitudinal MRI sequences for these models, as demonstrated here, resolves these challenges by allowing for accurate, reproducible, and non-invasive tracking of tumor burden.

While MRI has been previously employed in HCC models to verify tumor development, many studies have been performed in larger rodents (e.g. rats), did not reflect the etiological context of HCC (fibrosis or MASLD), used a contrast agent or reported a higher minimum detectable tumor size^26–28^. Here, we demonstrate that MRI is feasible in mice to detect liver tumors as small as 1 mm in diameter and without the need for a contrast agent, which improves animal welfare as no invasive procedures such as injections are necessary. In addition, our study uniquely integrates chronic liver disease with DEN-induced HCC, providing novel insights into this combined pathology.

Furthermore, we made important observations regarding the effect of immune checkpoint inhibition on multifocal orthotopic HCC growth. While MRI-based measurements revealed a significant reduction in the size of the largest tumors in αPD-L1-treated mice, the overall tumor burden remained comparable between treated and untreated groups. This indicates that immune checkpoint inhibition may selectively constrain the expansion of dominant lesions without substantially affecting total tumor volume. These findings highlight the limitations of traditional endpoint measurements like ex vivo-determined tumor size and underscore the value of MRI in capturing more nuanced treatment effects in complex, multilocular orthotopic tumor models by measuring tumor volume and overall tumor burden. These results should be interpreted as exploratory, given the limited sample size and the absence of a direct correlation between MRI-derived parameters and oncological markers of response. In the future, complementary immune profiling will be required to confirm these trends and to clarify the relationship between MRI-based tumor metrics and treatment efficacy.

The use of MRI in this context aligns with the aim of reducing preclinical animal numbers and suffering while increasing the accuracy and scientific benefit from conducted experiments^29^. Longitudinal tumor measurements allow to reduce the number of endpoints required for tumor growth assessment. This may particularly benefit immunotherapy studies, where treatment effects may be subtle or slow to manifest, and where a more comprehensive understanding of tumor kinetics can optimize therapeutic strategies. Furthermore, determining appropriate humane endpoints in the absence of non-invasive imaging can be challenging in orthotopic models. First, tumors located posteriorly may not be palpable until late tumor stages. Second, palpation may be compromised in those dietary models exhibiting abdominal/visceral obesity^30^. Consequently, long-term animal survival may insufficiently reflect the true oncological status and non-invasive MRI can be used to better standardize humane endpoints and prevent unnecessary animal suffering. It is important to note that in this study, the tumor-burden related humane endpoint was a tumor diameter of 15 mm due to legal animal welfare regulations, which led to a later discrepancy between the survival curves and the final tumor volume (i.e. actual tumor burden). This further highlights how MRI could be utilized to improve the definition of humane endpoints.

While this study provides valuable insight into the utility of MRI for monitoring tumor burden in experimental HCC, several limitations should be considered. DEN-induced tumors have well-defined histologic borders, which facilitate tumor identification on imaging^31^. In contrast, genetic tumor models, such as TAK1^LPC−KO^ and DEN, c-Myc, AlbLTαβ, and Mcl-1^Δhep^) display greater histologic and genomic heterogeneity, with less clearly defined tumor margins^12^. Therefore, applying our MRI-based approach to these models may require preliminary testing and protocol optimization. Furthermore, in large-scale experimental settings, the added time per animal, logistics and costs associated with MRI must be identified early and addressed with the support from funding agencies, experimental animal welfare organizations and 3R core facilities^32^. An important consideration for DEN models is that their reproducibility is dependent on strain, sex, age at injection and additional treatment, such as induction of fibrosis and dietary feeding. Therefore, detailed reporting of these factors in future studies is essential to maintain comparability. Finally, some end-point parameters such as liver weight were acquired at different timepoints in our study, depending on when the individual animal reached a humane endpoint, resulting in an overall low comparability between parameters obtained at euthanasia of long-term animals.

In conclusion, the present data hold implications for the design and conduction of murine HCC studies with high translational value. MRI-based tumor volume quantification allows to assess tumor growth dynamics longitudinally as well as track both large and small tumors individually, which may be useful to study mixed responses between different lesions. This may be impactful both from an animal welfare perspective in reducing animal numbers and suffering, but also by increasing the accuracy, reproducibility and objectivity of tumor load measurement by VOI calculation.

## Supplementary Information

Below is the link to the electronic supplementary material.

Supplementary Material 1

## Data Availability

The datasets generated analyzed during the current study are available from the corresponding author on reasonable request.

## Abbreviations

ALP: Alkaline phosphatase
ALT: Alanine aminotransferase
AST: Aspartate aminotransferase
BW: Bodyweight
CCl_4_: Carbon tetrachloride
DEN: Diethylnitrosamine
HCC: Hepatocellular carcinoma
H&E: Hematoxylin and eosin
LAGeSo: Berlin state office for health and social affairs
LDH: Lactate dehydrogenase
MASLD: Metabolic dysfunction-associated steatotic liver disease
MASH: Metabolic dysfunction-associated steatohepatitis
MRI: Magnetic resonance imaging
OTB: Overall tumor burden
αPD-L1: Anti-programmed death-ligand 1
ROI: Region of interest
SD: Standard deviation
TME: Tumor microenvironment
TR: Repetition time
VOI: Volume of interest
WD: Western-type diet
