# Supplementary material for: Quantification of overall tumor burden using longitudinal magnetic resonance imaging improves response assessment in orthotopic murine hepatocellular carcinoma models
Source: Sci Rep. 2026 Feb 5;16:5247. doi: 10.1038/s41598-026-38125-2 (PMC12881393; doi:10.1038/s41598-026-38125-2)
Supplement: Supplementary file 1 — Supplementary Material 1 [file 41598_2026_38125_MOESM1_ESM.pdf]

Supplementary Figure 1

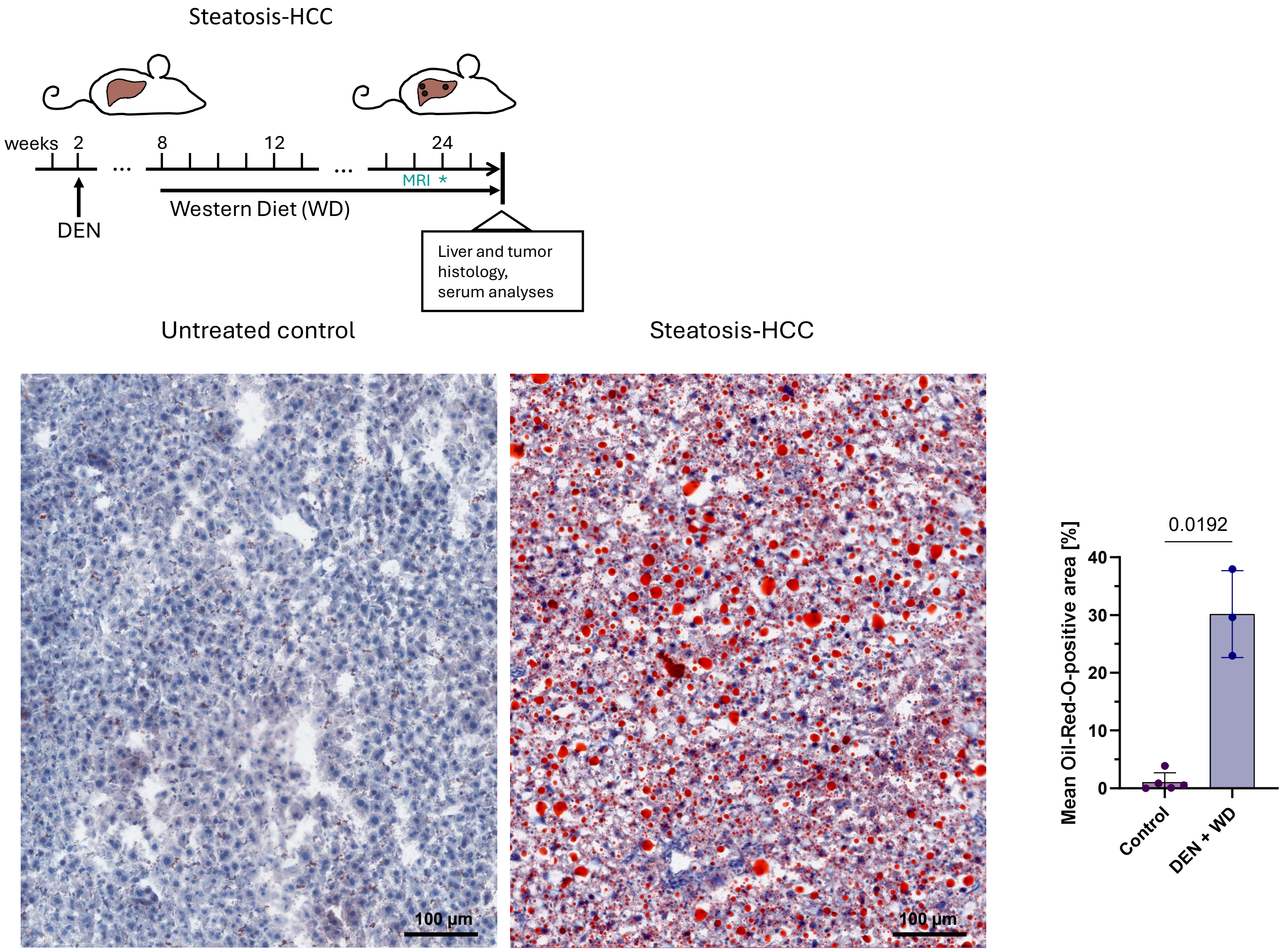

Supplementary Figure 1: Liver Steatosis is increased in the steatosis-HCC model

Oil red O Staining in untreated controls and the non-tumor area of MASLD-HCC. Scale bar = 100 µm. Unpaired t-test with Welch's correction.

Abbreviations: CCl_4, carbon tetrachloride, DEN, diethylnitrosamine, HCC, hepatocellular carcinoma, MRI, magnetic resonance imaging, WD, Western Diet

Supplementary Figure 2

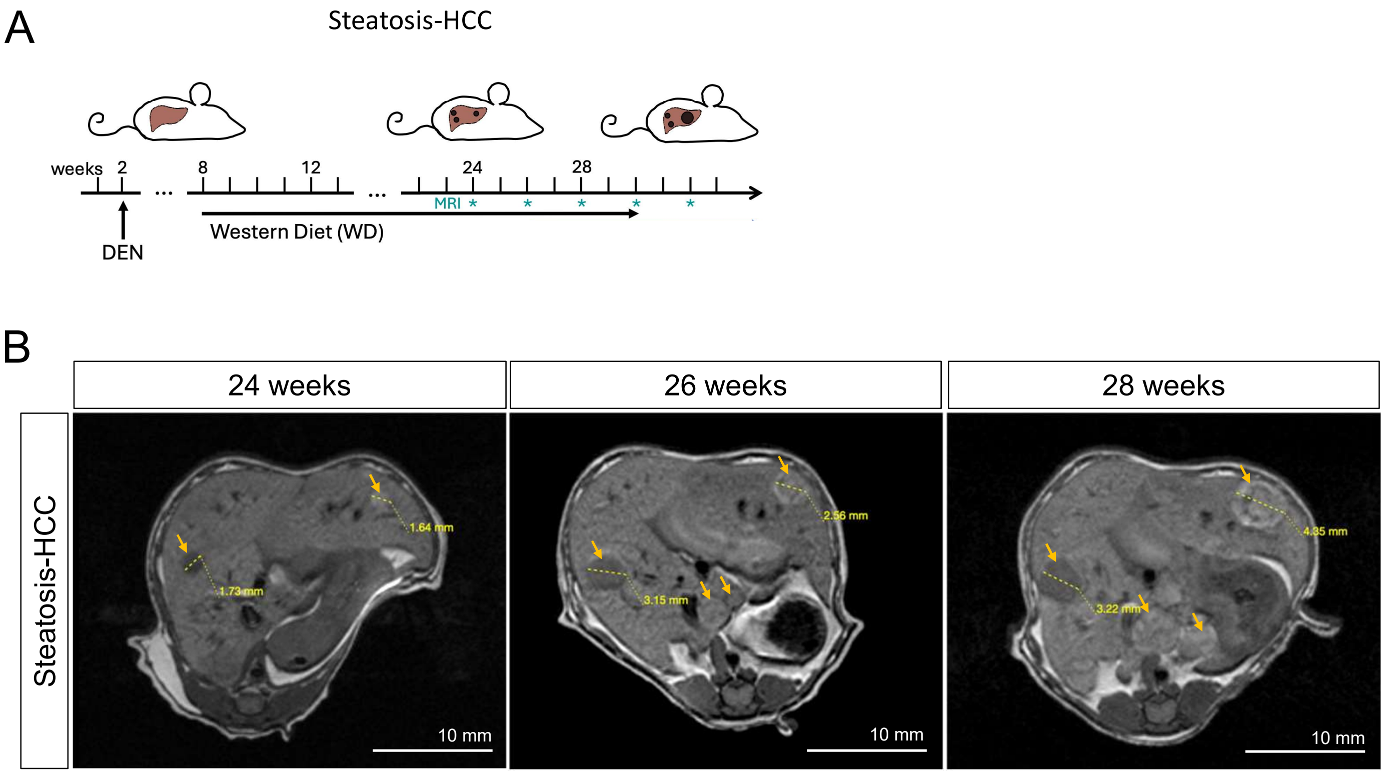

Supplementary Figure 2: MRI captures tumor heterogeneity, which is preserved during HCC progression.

(A) Schematic overview of the experimental timeline of the steatosis-HCC model.

(B) Representative T1-weighted axial MRI images from the same mouse at 24, 26 and 28 weeks of age. Yellow dashed lines indicate individual tumor nodules. Arrows indicate tumor nodules. Scale bar = 10 mm.

Abbreviations: DEN, diethylnitrosamine, HCC, hepatocellular carcinoma, WD, Western Diet

Supplementary Figure 3

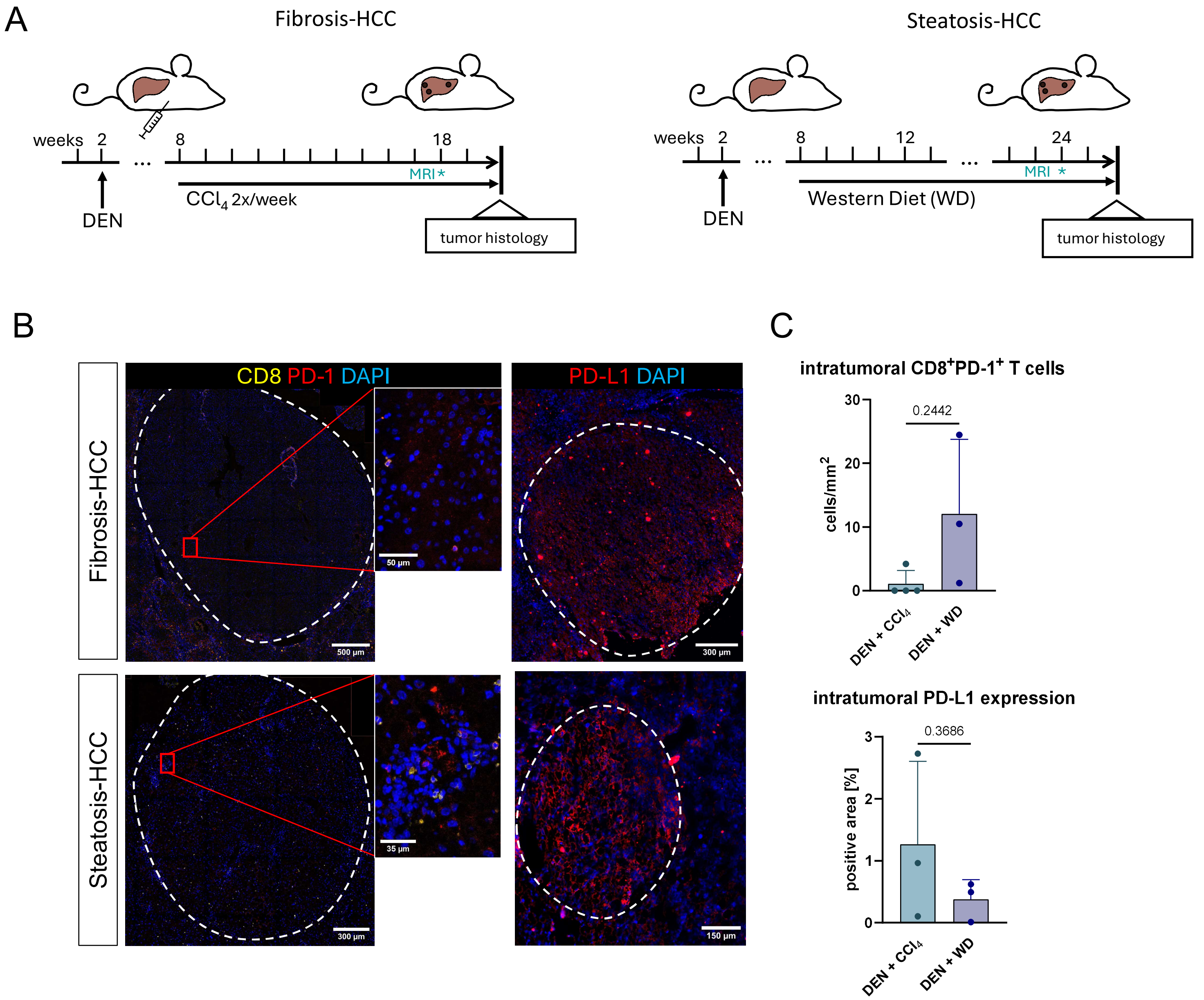

Supplementary Figure 3: HCC tumors express PD-L1 and harbor PD1+ CD8+T cells

(A) Schematic overview of the experimental timeline of both HCC models. (B) Representative immunofluorescent images of PD-1+CD8+ T cells (left) and PD-L1 expression (right) in tumor nodules. (C) Quantification of PD-1+CD8+ T cell numbers (top) and PD-L1 positive area (bottom) from stainings shown in B. Unpaired t-test with Welch´s correction.

Abbreviations: CCl_4, carbon tetrachloride, DEN, diethylnitrosamine, HCC, hepatocellular carcinoma, MRI, magnetic resonance imaging, WD, Western Diet

Supplementary Figure 4

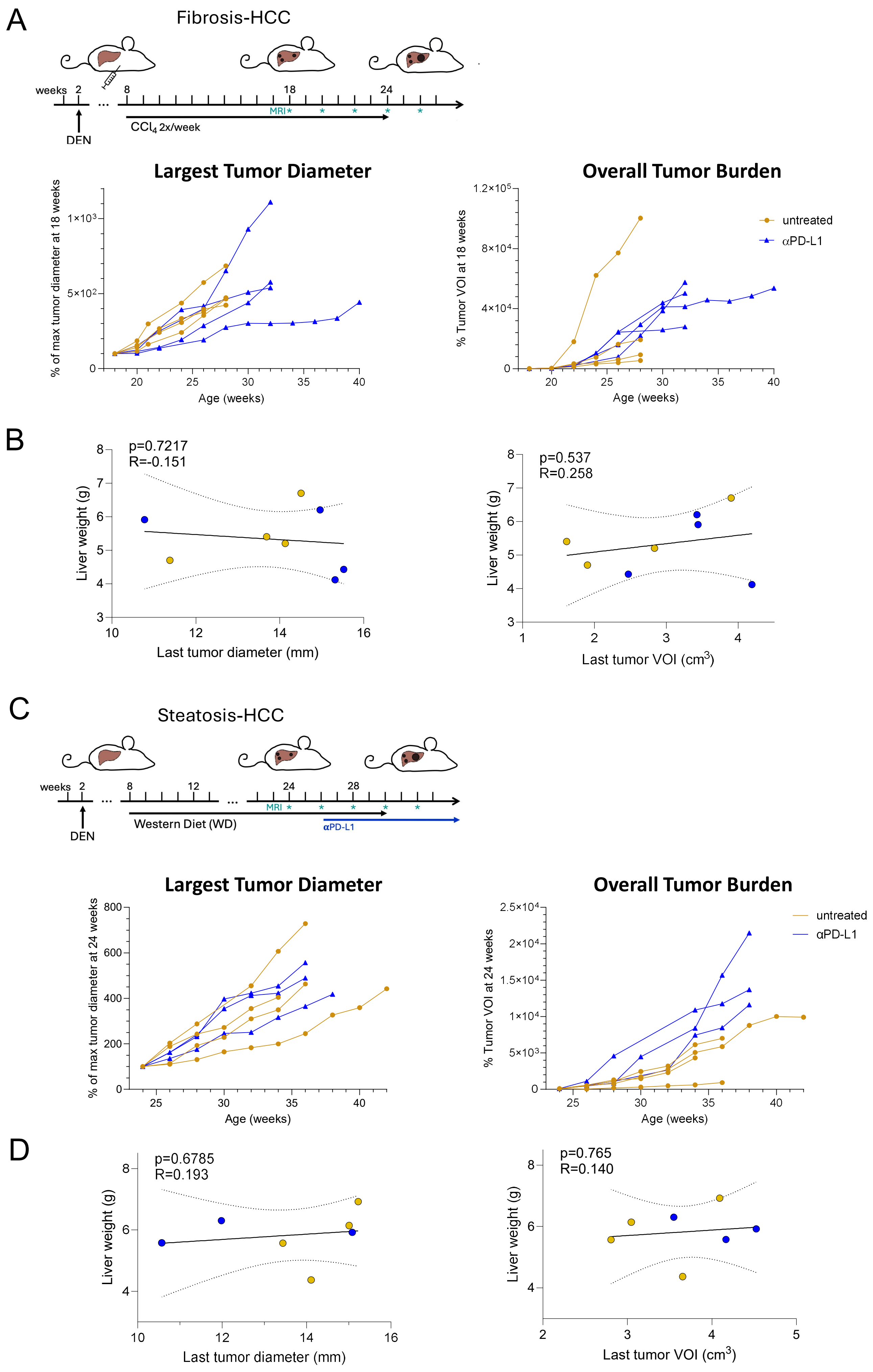

Supplementary Figure 4: Tumor growth expressed as % baseline.

(A) Experimental design of the fibrosis-HCC model and quantification of tumor progression (largest tumor diameter and overall tumor burden) over time expressed as % baseline. Each line represents one mouse. Graphs are truncated at 36 weeks. (B) Spearman correlation of MRI readouts with liver weight at euthanasia. (C) Experimental design of the steatosis-HCC model and quantification of tumor progression (largest tumor diameter and overall tumor burden) over time expressed as % baseline. Each line represents one mouse. (D) Spearman correlation of MRI readouts with liver weight at euthanasia.

Abbreviations: CCl_4, carbon tetrachloride, DEN, diethylnitrosamine, HCC, hepatocellular carcinoma, MRI, magnetic resonance imaging, αPD-L1, anti-programmed death-ligand 1, VOI, volume of interest
